# Upscaling and Risk Evaluation of the Synthesis of the 3,5-Diamino-1H-Pyrazole, Disperazol

**DOI:** 10.3390/ijms25126737

**Published:** 2024-06-19

**Authors:** Charlotte Uldahl Jansen, Katja Egeskov Grier, Jens Bo Andersen, Louise Dahl Hultqvist, Martin Nilsson, Claus Moser, Michael Graz, Tim Tolker-Nielsen, Michael Givskov, Katrine Qvortrup

**Affiliations:** 1 Department of Chemistry, Technical University of Denmark, DK-2800 Lyngby, Denmark; chajan@kemi.dtu.dk (C.U.J.);; 2 Costerton Biofilm Center, Department of Immunology and Microbiology, Faculty of Health and Medical Sciences, University of Copenhagen, DK-2200 Copenhagen, Denmark; 3 Department of Odontology, University of Copenhagen, DK-2200 Copenhagen, Denmark; 4 Department of Clinical Microbiology, Copenhagen University Hospital, Rigshospitalet, DK-2100 Copenhagen, Denmark

**Keywords:** biofilm, dispersal, pyrazole, c-di-GMP, large-scale synthesis, flow chemistry, safety, dust analysis, *Pseudomonas aeruginosa*, preclinical

## Abstract

This paper presents the work performed to transition a lab-scale synthesis (1 g) to a large-scale (400 g) synthesis of the 3-5-diamino-1H-Pyrazole Disperazol, a new pharmaceutical for treatment of antibiotic-resistant *Pseudomonas aeruginosa* biofilm infections. The potentially hazardous diazotisation step in the lab-scale synthesis was transformed to a safe and easy-to-handle flow chemistry step. Additionally, the paper presents an OSHA-recommended safety assessment of active compound **E**, as performed by Fauske and Associates, LLC, Burr Ridge, IL, USA.

## 1. Introduction

**Scheme 1:**
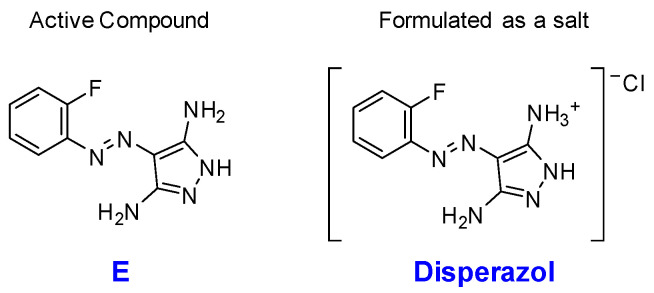
Chemical structure of active compound **E** and the formulated Disperazol.

In 2021, we presented [1] a potential new pharmaceutical **E** (see Scheme 1), developed for the treatment of antibiotic-resistant *Pseudomonas aeruginosa* biofilm infections. We showed that compound **E** interferes with c-di-GMP signalling to induce dispersal of *P. aeruginosa* biofilms [1], which allows the standard-of-care antibiotics to destroy the bacterial infection in vivo [2]. In the meantime, we published a synthetic route for compound **E** [3], as well as a study of its mode of action [4]. To increase water solubility, we formulated compound **E** as the HCl salt and have now named it Disperazol [2].

To further advance Disperazol as a novel pharmaceutical, a large-scale synthesis was pursued. This paper delineates the work performed to proceed from laboratory scale (lab scale) to large scale, more specifically, from a 1 g scale to a 400 g scale. Also, an evaluation of the physicochemical stability of active compound **E** is presented.

### 1.1. Safety Assessment of the Active Compound

Compound **E** has a relatively high ‘nitrogen-to-carbon’ ratio (N:C; 6:9, see Scheme 1), making it potentially explosive [5]; therefore, it is important to study the compound’s physicochemical stability before initiating a large-scale synthesis. While preliminary tests, including mechanical stress (friction by mortar and shock by wrench) and thermal stability (boiling in sulfuric acid) did not show any significant chemical degradation or decomposition, an extended investigation is needed. Table 1 presents the results from the ‘OSHA (Occupational Safety and Health Administration) Combustible Dust National Emphasis Program (NEP) Recommended Tests’ performed by Fauske and Associates, LLC.

The safety assessment concludes that active compound **E** is not sensitive to mechanical stress, shock or friction. In sum, it was stated that the *U.N. Manual of Tests and Criteria for Transportation of Dangerous Goods* considers compound **E** ‘safe to transport’. Looking at the minimum explosible concentration (MEC), minimum ignition energy (MIE), explosion sensitivity (ES), and ignition sensitivity (IS), it can be concluded that a dust cloud of **E** is sensitive to electrical sparks and therefore classified as an St3 explosive (deflagration index, Kst > 300). ES and IS for **E** are calculated using a minimum autoignition temperature (MIT) of 601 °C. These values classify **E** as a Class II dust according to the *National Electrical Code* ranking, and proper dust control and ventilation should be ensured in a large-scale facility. Dust clouds of **E** are not heat-sensitive below 600 °C, but show decomposition above 700 °C. When heated to above 700 °C, the monitored heat flow is positive, indicating that a reaction is taking place; this is probably a decomposition to CO_2_ and NO_X_.

### 1.2. Synthetic Route

**Scheme 2:**
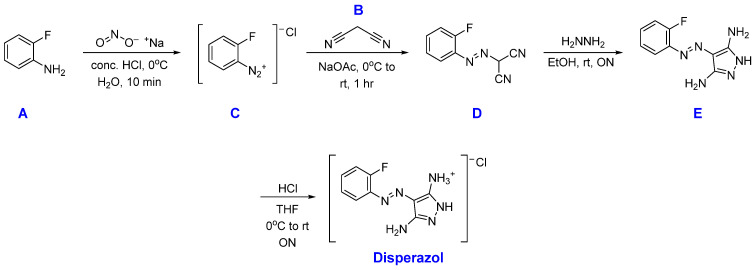
Lab-scale synthetic route [2,3], where rt is room temperature and ON is overnight.

During its initial development and optimisation, Disperazol was synthesised through a three-step synthesis [3], as shown in Scheme 2, without isolation of the diazonium ion **C**. Initially, the diazonium ion is formed from the aniline **A** and stabilised under cold acidic conditions. The diazotised aniline **C** is then quenched by the addition of malononitrile (**B**), which reacts to give the intermediate **D**. Intermediate **D** is isolated by precipitation and then dissolved in EtOH to undergo ring-closure upon the addition of hydrazine, leading to the formation of active compound **E**. The last step is the HCl-salt formation which provides Disperazol with improved water solubility [2].

## 2. Results and Discussion

It is important to consider physical parameters when scaling up a synthesis [6]. The surface-area-to-mass relationship, especially for heat/cold transfer, along with mixing, in addition to the chemical and physical properties, play important roles in organic chemistry and can be significantly different in a large-scale synthesis, compared to a batch reaction. Therefore, care should be taken when developing a large-scale process.

### 2.1. Step 1—Diazotisation

When scaling up a diazotisation reaction, the instability of the formed diazonium ion must be acknowledged. The current reaction involves the formation of the aromatic diazonium-ion **C**, which is unstable if not handled correctly. Upon decomposition, the diazonium-species rapidly loses N_2_ (gas), which can build pressure inside the reaction container [7]. Ensuring a low temperature (<5 °C) [8] is crucial for the safety of this step, which can be difficult in a large-scale production. Historically, most incidents with diazo coupling reactions have resulted from isolation and/or accumulation of a diazonium species for which decomposition can be caused by shock, friction, heat, light, or a number of other factors [8,9,10,11,12,13]. One of the main reasons for this limited stability is the low bond-strength between the diazo group and the aromatic ring [9]; therefore, both the substitution pattern and counterion will affect the stability [14]. In general, smaller counterions like chlorides and acetates have low stability, whereas larger counterions like tetrafluoroborates and tosylates provide higher stability [15].

**Scheme 3:**
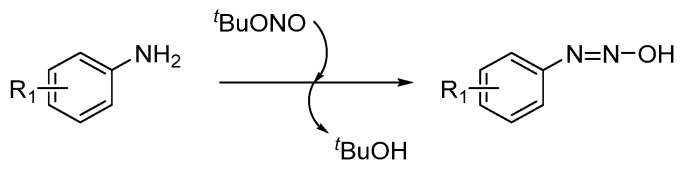
Formation of a hydroxydiazene intermediate [26,27].

To eliminate the potential danger of the diazotization step, we pursued the development of a safe and ‘easy to handle’ procedure to form the intermediate **D**. Inspired by the work of Jacq and Pasau [16], this involved the following: (1) Substituting *tert*-butyl nitrite (TBN) for sodium nitrite (NaNO_2_) as a safer diazonium reagent, which would also enable the reaction to be run safely at higher temperatures. (2) Transferring the first step of the synthesis from batch to flow synthesis, and thereby avoiding accumulation of the unstable intermediate **C**. TBN has been reported to be a mild, nonexplosive, and stable diazonium reagent [17,18,19,20] with a high safety profile in both batch and flow processes [16,21], and from small to bulk multikilogram scale [22,23]. Diazotization reactions using TBN have been run at temperatures ranging from room temperature to 80 °C [24,25]. The higher stability of the in situ formed diazonium species could arise from the lager counterion in *tert*-butyl oxide but it has also been suggested that a hydroxydiazene intermediate is formed (Scheme 3) [26,27].

Reactions that involve the formation of hazardous intermediates are advantageously run using flow chemistry, to ensure that the reactive hazardous species is continuously formed in small amounts. Immediate consumption will then eliminate the need for stockpiling [9,15,28,29]. As accumulation of large quantities of the hazardous compound is avoided; flow synthesis also eliminates the need for the cooling and even the heating of diazotization reactions has been performed safely [30]. The many advantages in flow synthesis provide an opportunity for both the academic and industrial chemical sectors to safely run chemical reactions with hazardous compounds. Flow chemistry is, for example, applied for diazotization reactions by established companies such as Lundbeck [31] and Pfizer [32].

**Scheme 4:**
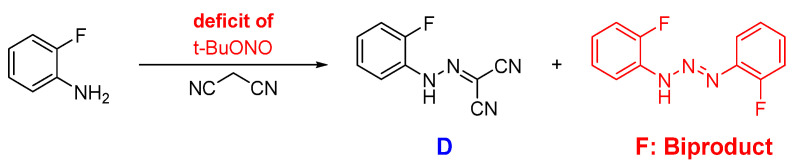
Reaction scheme for the formation of biproduct F under the condition of TBN being in deficit.

Inspired by Jacq and Pasau [16], we initiated our work, aiming towards establishing a flow synthesis of Disperazol by setting up the Uniqsis FlowSyn machine (Uniqsis Ltd., Cambridge, UK) at room temperature with a reagent ratio of 1:1:1.3 (aniline:malononitrile:TBN), an aniline concentration of 0.4 M, and a coil resident time of 5 min. However, these conditions resulted in both lower yields and the formation of impurities. Therefore, the flow reaction required some optimisation. One of the main impurities being formed was the biproduct **F**, resulting from the condensation of the aryl diazonium species with another aniline species; see Scheme 4. This was not observed under the original lab-scale synthesis and is most likely a result of changing the reaction conditions. The diazotisation reaction is no longer run under acidic conditions, which will make the aniline nucleophile. To optimize the reaction, a range of parameters was investigated; see Table 2 for a quick overview and SI for the extended table with specific details and purities for each experiment. By changing them one at a time, the effect of each parameter on the purity of the crude product was determined. All reactions were analysed with HPLC.

**Scheme 5:**
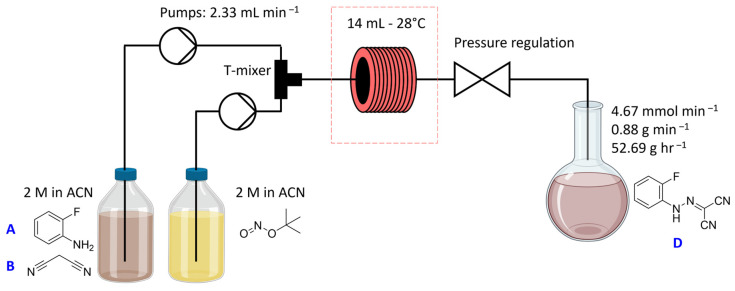
Schematic of the flow design for step 1. Made with BioRender (biorender.com) and PowerPoint (Microsoft 365).

Through the investigations it was found that a temperature of 28 °C in the reaction coil gave high product purity, with low reaction time (3 min) and with high concentrations of reactants (2 M), in a 1:1:1 ratio. After an extensive number of reactions (see Table 2), an optimised flow procedure was identified, allowing the formation of pure intermediate **D** in high yields in a short time. In the optimised flow design, see Scheme 5, the 2-fluoro aniline (**A**) and malononitrile (**B**) were mixed in one flask (ratio 1:1) in acetonitrile (ACN, 2 M), while the other flask contained the TBN dissolved in ACN (2 M). The two streams were telescoped in a T-mixer unit before being introduced to the PTFE coil reactor (14 mL) at 28 °C. Here the diazonium specie was formed in situ and instantly consumed by the malononitrile present in the same stream [16,25]. It was found that the reaction was slightly exothermic, so to ensure a constant temperature in the coil at 28 °C, a fan was placed next to the coil. This enabled the production of approximately 420 g (280 mmol) of **D** in 8 h.

### 2.2. Step 2—Ring Closure of Pyrazole

Steps 2 and 3 of the synthesis were both performed as batch chemistry. The second step of the small-scale synthesis proceeds by dissolving the intermediate **D** in ethanol (0.34 M), followed by the addition of hydrazine hydrate (1.2 equiv.). The mixture was allowed to stir overnight at room temperature, resulting in precipitation of active compound **E** [3]. To ensure full precipitation and thereby improve the yield on a larger scale, a series of solvents was investigated, as compound **E** is partly soluble in ethanol. The solvents investigated included ACN, water, acidic and basic aqueous solutions, diethyl ether, toluene, tetrahydrofuran (THF), heptane, dichloromethane (DCM), and acetone. From the solvent tests, it was found that **E** nicely precipitated in diethyl ether, while solubility of the intermediate **D** was achievable upon heating. Even though diethyl ether has a low flash point and is highly flammable, it was chosen as the solvent due to the ease of isolation of the pure product in high yields via filtration. When running the reaction at large scale, it was observed that the ring closure was rather exothermic. While this required the slow addition of hydrazine hydrate (1.15 equiv.), it was fortunately found to help in dissolving **D** without the need for external heating. Diethyl ether was found to be a good solvent with a concentration of intermediate **D** at 0.5 M. For step 2 the largest amount of compound **E** synthesised in one round was 652 g.

### 2.3. Step 3—Formulation as a Salt

To improve the water solubility of active compound **E**, it was formulated as a hydrochloride-salt [2], E•HCl, denominated Disperazol. For the lab-scale synthesis [2] of Disperazol, active compound **E** was dissolved in dry 1,4-dioxane (0.1 M), followed by the addition of a solution of HCl (4 M in dioxane, 5 equiv.) under a nitrogen atmosphere and the mixture was stirred overnight. For the large-scale synthesis, again, different solvents were investigated, as 1,4-dioxane is carcinogenic. Also, different reaction concentrations were tested, as large-scale reactions are preferably run at concentrations higher than 0.1 M to limit the reaction volume. The investigation included diethyl ether, acetone, ethyl acetate, and THF; THF was found to provide the most reliable results and a concentration of 0.2 M was found to result in good conversion and high purity. For the large-scale synthesis, compound **E** is dissolved in dry THF (0.2 M) under a nitrogen atmosphere with gentle heating to fully dissolve the active compound. The mixture is then cooled to 0 °C using an ice bath, before the addition of HCl (2 M in diethyl ether, 1 equiv.); afterwards, it is allowed to stir overnight at room temperature. The next day, the reaction mixture is cooled to 0 °C for two hours to ensure full product precipitation, before isolating the product by suction filtration. In case the precipitation does not start upon cooling, dry diethyl ether is added to start the precipitation. The largest scale synthesised formed 43 g of Disperazol (80%).

### 2.4. Summary

The synthetic route for Disperazol has successfully been scaled from a 9 mmol scale to a 3.6 mol scale with satisfying yields and easy purification. To eliminate the potential danger of the diazotization step, a flow-strategy was implemented and optimised, resulting in a clean reaction with good yields. Additionally, the physicochemical stability properties of active compound **E** were evaluated for both the solid powder and the dust particles, providing insights for a future implementation in an industrial production facility. It was found that the solid powder of **E** was non-explosive for all tested parameters.

## 3. Materials and Methods

Flow synthesis was performed on a Uniqsis FlowSyn System with FlowSyn Steel™, 316 L flow path, 2 × 10 mL/min, Pmax = 100 bar, Tmax = 260 °C and a 14 mL PTFE coil. The inner diameter of the coil tubing is 1.0 mm.

Here is the procedure for large-scale synthesis of Disperazol: For step 1, synthesis of **D**, the two solutions are prepared for the flow system. Flask A contains 2-fluoroaniline (CAS 348-54-9, 444.44 g, 1 equiv.), malononitrile (CAS 109-77-3, 264.39 g, 1 equiv.), and acetonitrile (75-05-8, fill up to 2000 mL). Flask B contains *tert*-butyl nitrite (540-80-7, 475.7 mL, 1 equiv.) and acetonitrile (75-05-8, fill up to 2000 mL). The flow system is configured as shown in Scheme 5.

For the reaction using FlowSyn, 99% of the prepared solutions (Flask A and B) is used (1980 mL), and the coil residence time is set to 3 min, giving a total flow rate of 4.67 mL/min. The coil temperature is set to 28 °C, and pre- and post-collect are set to 0.5 and 8.5 mL respectively. Additionally, a small fan is installed next to the coil to keep the temperature constant at 28 °C, given the slightly exothermic reaction; see the supporting information for details. Total operation time is approximately 14 h, without the final washing of the system. Then, the solvent is removed in vacuo to give the crude intermediate **D** in quantitative yield (approx. 745 g, 3.96 mol), which is used directly in the next step.

Step 2 is the synthesis of **E**, the ring-closure. The solid intermediate **D** from step 1 (745 g, 1 equiv.) is transferred to a conical flask (not round-bottomed) with a stir bar. Solvent (Et_2_O, CAS 60-29-7, 2.02 mL/mmol, corresponding to 8000 mL) is added to give a concentration of 0.5 M, and to the slurry is then added hydrazine hydrate (CAS 10217-52-4, 221.4 mL, 1.15 equiv.) dropwise under constant stirring, as the reaction is highly exothermic. Upon addition of hydrazine, the intermediate will fully dissolve, and the product will start to precipitate. When all hydrazine has been added, the reaction is left stirring overnight at room temperature. On the next day, the product is isolated by suction filter and washed with cold Et_2_O to give active compound E as an orange solid (652 g, 75%).

The third step is the synthesis of Disperazol, salt formation. For better water solubility, active compound E is formulated as a hydrochloride-salt. This is achieved by dissolving the E from step 2 (46 g, 1 equiv.) in dry THF (CAS 109-99-9, 0.2 M, 5.32 mL/mmol, corresponding to 1110 mL) under a nitrogen atmosphere, with gentle heating, to fully dissolve the active compound. The solution is then cooled to 0 °C in an ice bath and HCl (2 M in Et_2_O, CAS 7647-01-0, 105 mL, 1 equiv.) is slowly added. The reaction is allowed to heat to room temperature and left overnight. On the next day, the reaction mixture is again cooled to 0 °C for two hours. The product is isolated by suction filtration and washed with a small amount of cold Et_2_O, giving Disperazol (43 g, 80%). In case the salt does not precipitate, dry Et_2_O can be added to the reaction mixture before filtration.

## Figures and Tables

**Table 1:** OSHA-recommended safety assessment of active compound E, conducted by Fauske and Associates, LLC.

TEST	Compound E
Drop-weight impact/Fallhammer (bulk sample)	>100 J
Friction sensitivity (bulk sample)	>360 N
Explosion severity, ES; Ignition sensitivity, IS (dust cloud)	P_max_ = 9.6 bar (dP/dt)_max_ = 1128 bar/s K_max_ = 306 bar·m/s ES = 2.9 IS = 13.1
Minimum explosible concentration, MEC (dust cloud)	50–60 g/m^3^ Estimate: 56 g/m^3^
Minimum ignition energy, MIE (dust cloud)	3–10 mJ Estimate: 8 mJ
Minimum autoignition temperature, MIT (dust cloud)	>600 °C
Total combustible content (bulk sample)	100 wt% > 700 °C

**Table 2:** Parameters alternated during optimisation of the flow setup; see table with specifics for each experiment in the Supplementary Materials.

Parameters	Range Tested
Concentration of **A**	0.3 M, 0.4 M, 0.6 M, 0.8 M, 1.0 M, 2.0 M
Temperature	Room temperature (ranging from 23–26 °C), 28 °C, 30 °C
Coil resident time	2.5 min, 3 min, 4 min, 5 min, 6.5 min, 7.5 min, 8 min, 10 min, 12 min, 15 min
Reagents ratio (**A**:**B**:TBN)	1:1:1, 1:1:1.3, 1:1:1.7, 1:1:2, 1:1:2.3, 1:1.1:1, 1:1.2:1, 1:1.3:2, 1:1.3:1.3, 1:1.5:1.3, 1:2:1.3

## Data Availability

The original contributions presented in the study are included in the article/Supplementary Material, further inquiries can be directed to the corresponding author.

## Supplementary Materials

The supporting information can be downloaded at: https://www.mdpi.com/article/10.3390/ijms25126737/s1.

## References

[1] Andersen J.B., Hultqvist L.D., Jansen C.U., Jakobsen T.H., Nilsson M., Rybtke M., Uhd J., Fritz B.G., Seifert R., Berthelsen J. (2021). Identification of small molecules that interfere with c-di-GMP signaling and induce dispersal of *Pseudomonas aeruginosa* biofilms. Npj Biofilms Microbiomes.

[2] Hultqvist L.D., Andersen J.B., Nilsson C.M., Jansen C.U., Rybtke M., Jakobsen T.H., Nielsen T.E., Qvortrup K., Moser C., Graz M. (2024). High efficacy treatment of murine *Pseudomonas aeruginosa* catheter-associated urinary tract infections using the c-di-GMP modulating anti-biofilm compound Disperazol in combination with ciprofloxacin. Antimicrob. Agents Chemother..

[3] Jansen C.U., Uhd J., Andersen J.B., Hultqvist L.D., Jakobsen T.H., Nilsson M., Nielsen T.E., Givskov M., Tolker-Nielsen T., Qvortrup K.M. (2021). SAR study of 4-arylazo-3,5-diamino-1 *H*-pyrazoles: Identification of small molecules that induce dispersal of *Pseudomonas aeruginosa* biofilms. RSC Med. Chem..

[4] Manner C., Dias Teixeira R., Saha D., Kaczmarczyk A., Zemp R., Wyss F., Jaeger T., Laventie B.-J., Boyer S., Malone J.G. (2023). A genetic switch controls *Pseudomonas aeruginosa* surface colonization. Nat. Microbiol..

[5] Tarselli M.A. (2012). Life and death with nitrogen. Nat. Chem..

[6] Warawdekar M.G. (2016). Challenges in Scale-Up of Specialty Chemicals—A Development Chemist’s Perspective. Industrial Catalytic Processes for Fine and Specialty Chemicals.

[7] Partington S., Waldram S.P. (2002). Runaway Reaction during Production of an Azo Dye Intermediate. Process Saf. Environ. Prot..

[8] Sheng M., Frurip D., Gorman D. (2015). Reactive chemical hazards of diazonium salts. J. Loss Prev. Process Ind..

[9] Filimonov V.D., Krasnokutskaya E.A., Bondarev A.A., Chehimi M.M., Pinson J., Mousli F. (2022). Structures, Stability, and Safety of Diazonium Salts. Aryl Diazonium Salts and Related Compounds.

[10] Bretherick L., Urben P.G. (2017). Bretherick’s Handbook of Reactive Chemical Hazards: An Indexed Guide to Published Data.

[11] Ullrich R., Grewer T. (1993). Decomposition of aromatic diazonium compounds. Thermochim. Acta.

[12] Kittsley S. (1971). Need for minimum standards in evaluating doctoral programs. J. Chem. Educ..

[13] Firth J.D., Fairlamb I.J.S. (2020). A Need for Caution in the Preparation and Application of Synthetically Versatile Aryl Diazonium Tetrafluoroborate Salts. Org. Lett..

[14] Bondarev A.A., Naumov E.V., Kassanova A.Z., Krasnokutskaya E.A., Stankevich K.S., Filimonov V.D. (2019). First Study of the Thermal and Storage Stability of Arenediazonium Triflates Comparing to 4-Nitrobenzenediazonium Tosylate and Tetrafluoroborate by Calorimetric Methods. Org. Process Res. Dev..

[15] Deadman B.J., Collins S.G., Maguire A.R. (2015). Taming Hazardous Chemistry in Flow: The Continuous Processing of Diazo and Diazonium Compounds. Chem.-Eur. J..

[16] Jacq J., Pasau P. (2014). Multistep Flow Synthesis of 5-Amino-2-aryl-2 *H*-[1,2,3]-triazole-4-carbonitriles. Chem.-Eur. J..

[17] Crisóstomo F.P., Martín T., Carrillo R. (2014). Ascorbic Acid as an Initiator for the Direct C-H Arylation of (Hetero)arenes with Anilines Nitrosated In Situ. Angew. Chem. Int. Ed..

[18] Qiu D., Meng H., Jin L., Wang S., Tang S., Wang X., Mo F., Zhang Y., Wang J. (2013). Synthesis of Aryl Trimethylstannanes from Aryl Amines: A Sandmeyer-Type Stannylation Reaction. Angew. Chem. Int. Ed..

[19] Chakraborty A., Jana S., Kibriya G., Dey A., Hajra A. (2016). tert-Butyl nitrite mediated azo coupling between anilines and imidazoheterocycles. RSC Adv..

[20] He L., Qiu G., Gao Y., Wu J. (2014). Removal of amino groups from anilines through diazonium salt-based reactions. Org. Biomol. Chem..

[21] Hu T., Baxendale I., Baumann M. (2016). Exploring Flow Procedures for Diazonium Formation. Molecules.

[22] Mihelač M., Siljanovska A., Košmrlj J. (2021). A convenient approach to arenediazonium tosylates. Dyes Pigment..

[23] Oger N., d’Halluin M., Le Grognec E., Felpin F.-X. (2014). Using Aryl Diazonium Salts in Palladium-Catalyzed Reactions under Safer Conditions. Org. Process Res. Dev..

[24] Mo F., Jiang Y., Qiu D., Zhang Y., Wang J. (2010). Direct Conversion of Arylamines to Pinacol Boronates: A Metal-Free Borylation Process. Angew. Chem. Int. Ed..

[25] Oger N., Le Grognec E., Felpin F.-X. (2015). Handling diazonium salts in flow for organic and material chemistry. Org. Chem. Front..

[26] Callonnec F.L., Fouquet E., Felpin F.-X. (2011). Unprecedented Substoichiometric Use of Hazardous Aryl Diazonium Salts in the Heck-Matsuda Reaction via a Double Catalytic Cycle. Org. Lett..

[27] Susperregui N., Miqueu K., Sotiropoulos J.-M., Le Callonnec F., Fouquet E., Felpin F.-X. (2012). Sustainable Heck-Matsuda Reaction with Catalytic Amounts of Diazonium Salts: An Experimental and Theoretical Study. Chem.-Eur. J..

[28] Ahmed-Omer B., Barrow D.A., Wirth T. (2009). Heck reactions using segmented flow conditions. Tetrahedron Lett..

[29] Movsisyan M., Delbeke E.I.P., Berton J.K.E.T., Battilocchio C., Ley S.V., Stevens C.V. (2016). Taming hazardous chemistry by continuous flow technology. Chem. Soc. Rev..

[30] Smith C.J., Smith C.D., Nikbin N., Ley S.V., Baxendale I.R. (2011). Flow synthesis of organic azides and the multistep synthesis of imines and amines using a new monolithic triphenylphosphine reagent. Org. Biomol. Chem..

[31] Nielsen M.A., Nielsen M.K., Pittelkow T. (2004). Scale-Up and Safety Evaluation of a Sandmeyer Reaction. Org. Process Res. Dev..

[32] Li B., Widlicka D., Boucher S., Hayward C., Lucas J., Murray J.C., O’Neil B.T., Pfisterer D., Samp L., VanAlsten J. (2012). Telescoped Flow Process for the Syntheses of *N*-Aryl Pyrazoles. Org. Process Res. Dev..

