# Supplementary material for: Upscaling and Risk Evaluation of the Synthesis of the 3,5-Diamino-1H-Pyrazole, Disperazol
Source: Int J Mol Sci. 2024 Jun 19;25(12):6737. doi: 10.3390/ijms25126737 (PMC11203405; doi:10.3390/ijms25126737)
Supplement: Supplementary file 1 [file ijms-25-06737-s001.zip › ijms-3048741-supplementary.pdf]

International Journal of
Molecular Sciences

MDPI

Article

Upscaling and Risk Evaluation of the Synthesis of the 3,5-Diamino-1H-Pyrazole, Disperazol

Charlotte Uldahl Jansen [1], Katja Egeskov Grier [1], Jens Bo Andersen [2], Louise Dahl Hultqvist [2], Martin Nilsson [3], Claus Moser [2,4], Michael Graz [2], Tim Tolker-Nielsen [2], Michael Givskov [2] and Katrine Qvortrup [1,*]

1 Department of Chemistry, Technical University of Denmark, DK-2800 Lyngby, Denmark
2 Costerton Biofilm Center, Department of Immunology and Microbiology, Faculty of Health and Medical Sciences, University of Copenhagen, DK-2200 Copenhagen, Denmark
3 Department of Odontology, University of Copenhagen, DK-2200 Copenhagen, Denmark
4 Department of Clinical Microbiology, Copenhagen University Hospital, Rigshospitalet, DK-2100 Copenhagen, Denmark
* Correspondence: kaqvo@kemi.dtu.dk

Supplementary Materials

General Methodologies

Spectroscopic data

Table of flow conditions tested

Photos of the large scale setups

General Methodologies

All solvents were of HPLC quality from either Sigma-Aldrich or VWR Chemicals these and other commercially available reagents were used without further purification. The dry solvents DCM, Et2O, ACN and DMF were obtained from the in-house PureSolveTM MD-7 Solvent Purification System, from Innovative Technology were Al_2O_3 was used as the stationary phase. For dry reaction was glassware was flamedried, and cooled under dry N_2 atmosphere before use. Room temperature is approximately 22°C. Chemicals have been bought from Sigma-Aldrich, Combi-Blocks, Fluorochem and Fisher Scientific.

^{1}H-NMR, ^{13}C-NMR, COSY, spectras were recorded on Bruker Ascend spectrometer with a Prodigy cryoprobe operating at 400 MHz or 800 MHz for ^{1}HNMR and 101 MHz for ^{13}C-NMR. The specific deuterated solvent is stated for each compound. Chemical shifts (δ) are reported in ppm downfield from TMS (= 0) using solvent resonance as the internal standard (dimethylsulfoxide-d6, ^{1}H: 2.50 ppm, ^{13}C: 39.52 ppm). Coupling constants (J) are reported in Hz and the field is reported in each case. Multiplicities are reported as singlet (s), broad singlet (br. s), doublet (d), doublet of doublets (dd), doublet of triplets (dt), doublet of doublet of doublets (ddd), doublet of doublet of triplets (ddt), triplet (t), triplet of doublets (td), quartet (q), pentet (p), septet (sep) and multiplets (m).

Evaporation of the solvents were performed using a Heidolph Laborota 4000 efficient under reduced pressure (in vacuo) at different temperatures depending on the boiling point of the solvents.

Melting point was measured on a Stuart SMP30 and capillary tubes from VITREX (1.50x2.00x100 mm, one end closed).

N_2 atmosphere was used in experiments for obtaining an inert atmosphere in reactions that would otherwise react with water resulting in undesired side-reactions. N_2 atmosphere was achieved by applying a rubber septum to the reagent flask and hereafter adding a constant N_2 inlet through a Schlenk line.

HPLC was performed on a Waters e2695 Separations Module, with a EC 160/4.6 NUCLEOSIL 100-6 C18 coloum. The run was designed as: Solvent A – 0.1% HCOOH in water, Solvent B – 0.1% HCOOH in ACN. Gradient: 5% B, hold 2 min., gradient: 5% B to 70% B in 8 min., gradient: 70% B, hold 2 min., gradient: 70% B to 100% B in 8 min., gradient: 100% B, hold 2 min., gradient: 100% B to 5% B in 1 min., hold 2 min. Total run time – 25 min.

Spectroscopic data of intermediate D

HPLC

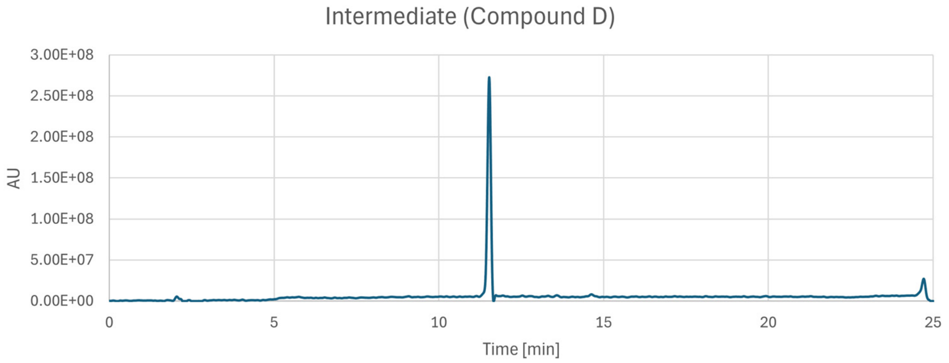

NMR: ^{1}H NMR (400 MHz, DMSO) δ 7.52 (td, *J* = 7.9, 2.5 Hz, 1H), 7.40 – 7.23 (m, 3H). ^{13}C NMR (101 MHz, DMSO) δ 152.89 (d, *J* = 248.7 Hz), 128.03 (d, *J* = 7.4 Hz), 125.76 (d, *J* = 3.7 Hz), 120.99, 117.20, 117.02, 114.68, 110.12, 86.99 (splitting due to F).

Melting point: 92±3°C (based on three mesurements).

Spectroscopic data of the active compound E

HPLC

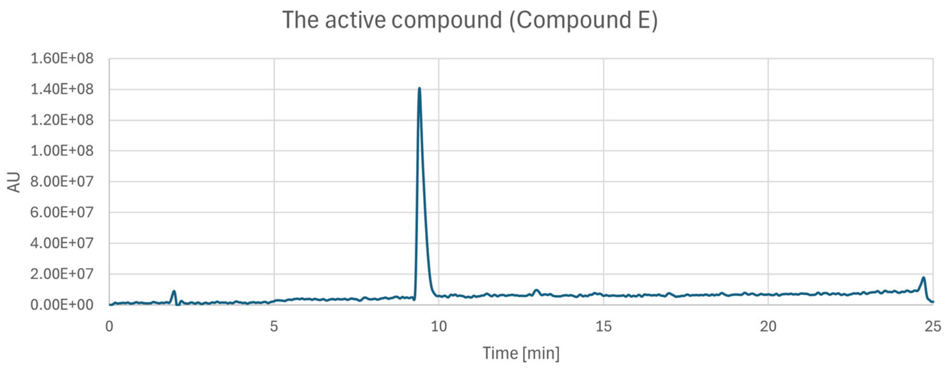

NMR: ^{1}H NMR (400 MHz, DMSO) δ 10.36 (s, 1H), 7.81 (td, *J* = 8.0, 2.1 Hz, 1H), 7.30 – 7.13 (m, 3H), 6.43 (s, 2H), 6.03 – 5.98 (m, 2H). ^{13}C NMR (101 MHz, DMSO) δ 157.50 (d, *J* = 249.0 Hz), 141.80, 141.74, 127.83 (d, *J* = 8.0 Hz), 124.84 (d, *J* = 3.4 Hz), 117.33, 116.70 (d, *J* = 19.8 Hz), 116.31, 98.06 (splitting due to F).

Melting point: 226±4°C (based on three mesurements).

Spectroscopic data of disperazol

HPLC

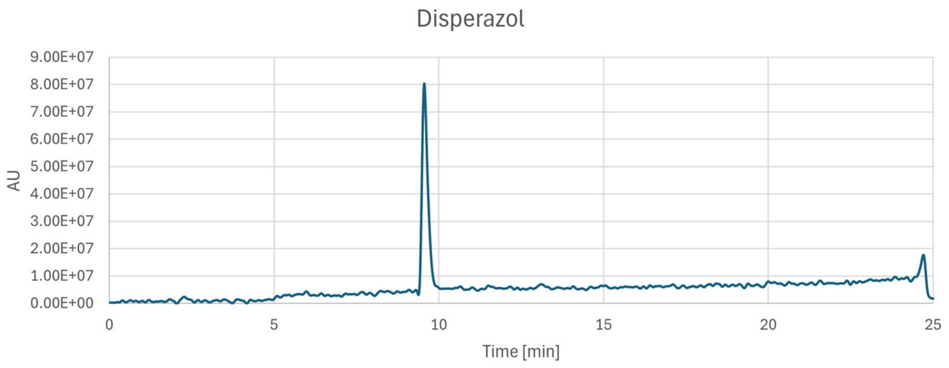

NMR: ^{1}H NMR (400 MHz, D_2O) δ 7.12 (dt, *J* = 19.1, 7.1 Hz, 2H), 6.98 – 6.85 (m, 2H). ^{13}C NMR (101 MHz, D_2O) δ 158.06 (d, *J* = 252.3 Hz), 139.07, 139.01, 130.58 (d, *J* = 8.4 Hz), 124.25, 116.28, 116.08, 115.93, 109.81 (splitting due to F).

Melting point: 205±3°C decomposition followed by complte melting at 215°C (based on three mesurements).

Table for all the conditions investigated during optimisation. Note that the yields reported are based on HPLC/LCMS and only relates to product compared to biproduct. Evidently experience in using the FlowSyn also improved yield. 2-Fluoroaniline (**A**), malononitrile (**B**), tertbutyl nitrite (**TBN**), RT = room temperature (approx. 23°C), *Product yield by HPLC compared to biproduct **F**.

**Entry**	**Conc. of A**	**Ratio (A:B:TBN)**	**Coil time**	**Temperature**	**Scale (A)**	**Product yield***	**Comment**
1	0.4 M	1:1:1.3	5 min	RT	1 g	68%	
2	0.3 M	1:1:1.3	5 min	RT	1 g	61%	
3	0.4 M	1:1:2	5 min	RT	1 g	85%	
4	0.6 M	1:1:2	5 min	RT	1.5 g	90%	
5	0.4 M	1:1:1.2	10 min	RT	1 g	74%	
6	0.4 M	1:1:1.3	2.5 min	RT	1 g	53%	
7	0.6 M	1:1:1.3	5 min	RT	1.5 g	80%	
8	0.4 M	1:1.3:1.3	5 min	RT	1 g	84%	
9	0.8 M	1:1:1.3	5 min	RT	2 g	88%	
10	0.8 M	1:1:2	5 min	RT (27°C)	2 g	95%	
11	0.8 M	1:1.3:2	5 min	RT	2 g	100%	
12	0.4 M	1:1:1.7	5 min	RT	1 g	93%	
13	0.4 M	1:1:2.3	5 min	RT	1 g	95%	
14	0.4 M	1:1.5:1.3	5 min	RT	1 g	89%	
15	0.4 M	1:2:1.3	5 min	RT	1 g	93%	
16 (re 3)	0.4 M	1:1:2	5 min	RT	1 g	94%	Experience
17	0.8 M	1:1.3:2	5 min	RT (>23°C)	1 g	92%	
18	1.0 M	1:1:1.3	5 min	RT (>23°C)	1 g	94%	
19	0.4 M	1:1:1.3	15 min	RT	1 g	93%	
20	0.4 M	1:1:1.3	7.5 min	RT	1 g	98%	
21	0.4 M	1:1:1.3	4 min	RT	1 g	78%	
22	0.4 M	1:1:1.3	6.5 min	RT	1 g	90%	
23 (re 1)	0.4 M	1:1:1.3	5 min	RT	1 g	83%	Experience
24	0.4 M	1:1:1.3	8 min	RT	1 g	100%	
25 (re 5)	0.4 M	1:1:1.3	10 min	RT	1 g	100%	Experience
26	0.4 M	1:1:1.3	12 min	RT	1 g	100%	
27 (re 1)	0.4 M	1:1:1.3	5 min	RT	1 g	100%	Experience
28	0.6 M	1:1:1.3	5 min	RT	1g	100%	Flasks A:B mixed in 1:2 ratio
29	0.3 M	1:1:1.3	5 min	RT	1 g	100%	Flasks A:B mixed in 2:1 ratio
30	1.0 M	1:1:1.3	5 min	RT	2 g	100%	
31	1.0 M	1:1:1	3 min	28°C	4 g	100%	
32	1.0 M	1:1:1	3 min	30°C	4 g	100%	
33	1.0 M	1:1.1:1	3 min	28°C	2 g	100%	
34	1.0 M	1:1.2:1	3 min	28°C	2 g	100%	
35	1.0 M	1:1.2:1	3 min	28°C	2 g	100%	Collect in fractions from flow
36	1.0 M	1:1:1	3 min	28°C	15 g	100%	Go up in scale
37	2.0 M	1:1:1	3 min	28°C	15 g	100%	Go up in conc.
38	2.0 M	1:1:1	3 min	28°C	22.23 g	100%	Wrong settings on flow
39	2.0 M	1:1:1	3 min	28°C	22.23 g	100%	Go up in scale
40	2.0 M	1:1:1	3 min	28°C	22.23 g	100%	Different supplier of A
41	2.0 M	1:1:1	3 min	28°C	444.44 g	100%	Final large scale

Photos of the large-scale synthesis

Step 1: FlowSyn setup	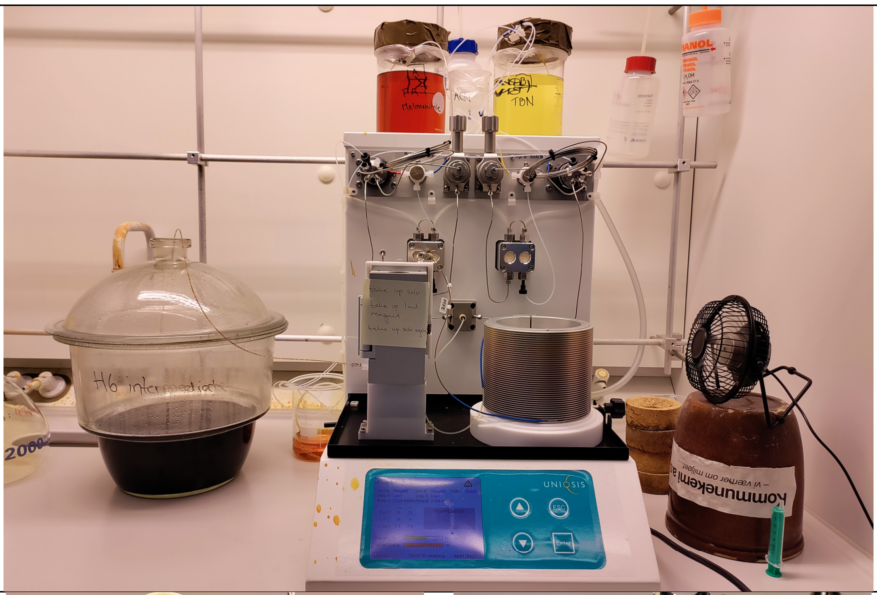
Step 2: Ring closure	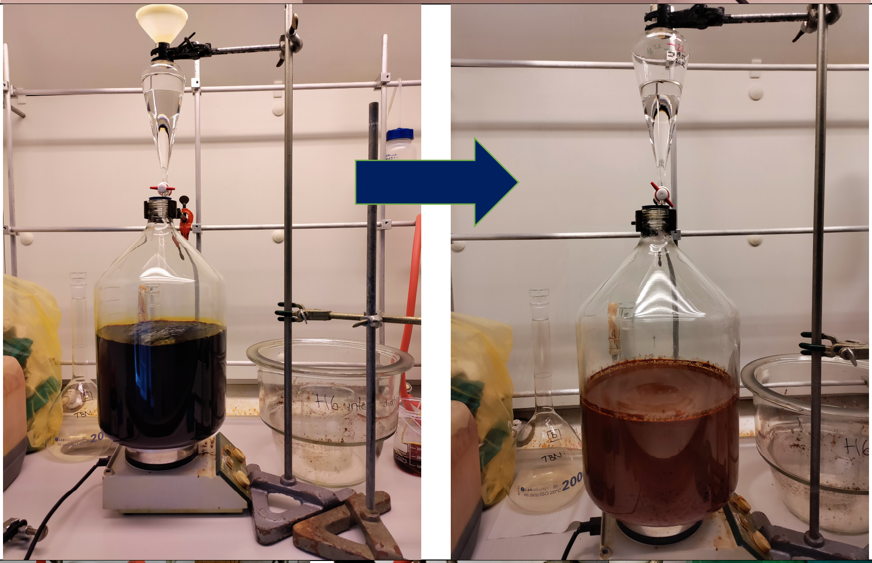
Step 3: Formulation as salt	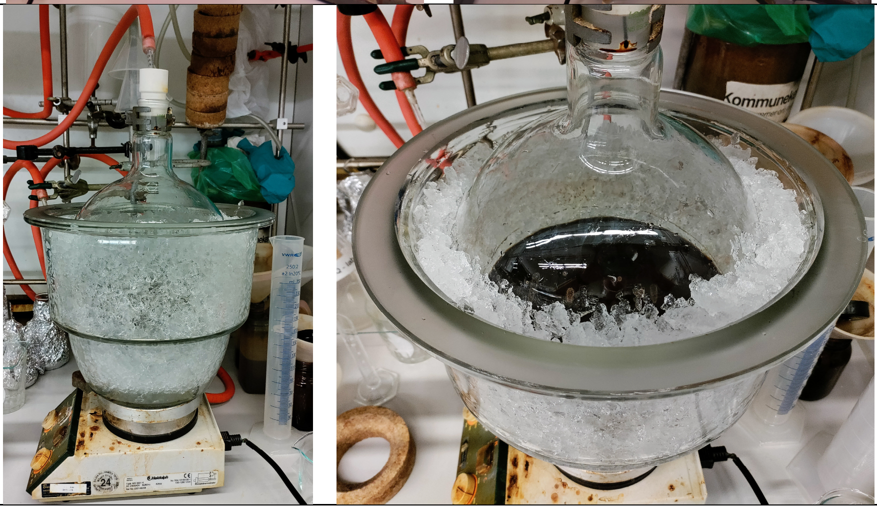
